# Using the health beliefs model to implement mobile puberty health education in Iranian adolescent boys: a randomized controlled trial

**DOI:** 10.3389/fpubh.2024.1175262

**Published:** 2024-02-08

**Authors:** Arash Salahshouri, Parvaneh Raisi-Philabadi, Saeed Ghanbari, Lar Stein, Marzieh Araban

**Affiliations:** ^1^Department of Health Education and Promotion, School of Public Health, Ahvaz Jundishapur University of Medical Sciences, Ahvaz, Iran; ^2^Department of Epidemiology and Biostatistics, School of Public Health, Ahvaz Jundishapur University of Medical Sciences, Ahvaz, Iran; ^3^Department of Psychology, University of Rhode Island, South Kingstown, RI, United States; ^4^Adjunct Research Faculty, Social and Behavioral Sciences, Brown University, Providence, RI, United States; ^5^Department of Behavioral Healthcare, Developmental Disabilities & Hospitals, Cranston, RI, United States; ^6^Menopause Andropause Research Center, Ahvaz Jundishapur University of Medical Sciences, Ahvaz, Iran

**Keywords:** adolescent males, health education, puberty, mobile health, health belief model

## Abstract

**Introduction:**

Given boys' low health knowledge and their unhealthy behavior during puberty, which can cause many physical, mental, and psychological problems, it is important to prevent these complications. This study was therefore aimed to determine the efficacy of a mobile health educational intervention based on the Health Beliefs Model (HBM) on Iranian adolescent boys.

**Materials and methods:**

This randomized controlled trial involved junior high school boys (*n* = 148) in Iran studying during the 2020–2021 school year. Educational content concerning healthy behaviors during puberty (e.g., the importance of bathing) was developed based on HBM and sent to the intervention group via mobile phone. HBM addresses multiple factors (e.g., perceived disease risk) that explain health behaviors. The intervention was delivered in five sessions over four weeks using real-time Internet communication and texting. The control group did not receive any intervention. One school was randomly selected from each of the four districts of the study site. The schools were then randomized into intervention and control groups. The boys were then randomly selected from each school to participate in the study. Data collected at baseline and 2-month follow-up assessments included demographic information, health knowledge (e.g., physical changes during puberty), health behaviors (e.g., bathing), and HBM constructs (e.g., self-efficacy to perform healthy behaviors). Data analysis was done using the chi-square, independent and paired *t-*tests, and analysis of covariance (ANCOVA).

**Results:**

The two groups did not differ in terms of demographic characteristics. Before the intervention, the two groups were slightly different in terms of knowledge, health behavior, and HBM constructs. Following the intervention, the scores of the intervention group improved significantly (*p* < 0.05). After adjusting for pre-intervention knowledge, HBM, and health behavior scores, the intervention group remained superior to the control group in terms of improvement of knowledge, HBM constructs, and healthy behaviors (*p* < 0.05). Effect sizes ranged from medium to large (0.25–0.86).

**Conclusion:**

Mobile phone education based on the HBM is efficacious in encouraging healthy behavior in boys during puberty. Organizations interested in encouraging healthy behaviors in boys should consider the use of such a program.

## 1 Introduction

Adolescence is an important period of life (1–3) as the individual transitions from childhood to adulthood (4–6). This period is characterized by many physical, emotional, and social changes (2, 4, 6, 7). Furthermore, during adolescence, individuals are at increased risk for mental health problems, which are probably associated with related hormonal changes (8, 9). Puberty, which includes the final years of childhood and the early years of adolescence (4), is associated with the release of puberty hormones, marking the beginning of sexual maturation. During this period, there is an increase in physical stature and metabolism, and secondary sexual characteristics emerge (7–10).

Many health habits are formed during adolescence, and this has important implications for health in adulthood (11). Moreover, physical changes during puberty can exacerbate low self-confidence and increase anxiety (12). Previous studies on Iranian girls indicate that many lack awareness regarding menstrual hygiene and are in need of education (3, 13, 14). In other studies, more than half of Iranian boys were found to be unaware of the signs of puberty, behaviors to foster physical and sexual health, and psychological changes during adolescence (12, 15). Adolescent boys have been reported to have unmet educational and counseling needs regarding adaptation to puberty changes and sexual orientation (16). In one study, for example, although a positive and significant correlation was observed between the health behaviors of adolescent boys and awareness of puberty changes, 69.81% of the studied boys lacked awareness of puberty and related health behaviors, and 87% did not have access to suitable educational resources (17).

Educating adolescents about changes associated with puberty reduces misinformation, risky behaviors, and diseases in adulthood (18–21). Empowering adolescents to improve their sexual health may not only help them avoid risky sexual behaviors and sexually transmitted infections but also encourage them to act responsibly in their sexual relationships (22). It is necessary to apply effective and age-appropriate strategies to educate adolescents regarding reproductive health and their changing bodies (23). Unfortunately, health education for Iranian boys has been a neglected topic in recent years (15).

Health education, improving health knowledge, changing attitudes toward health, and adopting healthy behaviors to prevent health problems can play an important role in promoting health and reducing disease (15, 24, 25). Health education programs should be theoretically informed and address the multiple factors that impact health behavior change (26). The Health Beliefs Model (HBM) addresses multiple factors (e.g., perceived disease risk, efficacy to perform behaviors to enhance or maintain health, etc.) that explain health behaviors (27). HBM is used to determine the relationship between beliefs and health behaviors and explains the reason why people perform or do not perform preventive health behaviors (15, 28).

According to the HBM (27), to take preventive actions, individuals must feel threatened by the problem (perceived susceptibility), understand the seriousness (perceived severity) of the risk, positively assess the benefits of preventive behavior, perceive relatively minimal obstacles (i.e., barriers) to change, have confidence in the ability to successfully make a change (i.e., self-efficacy), and be triggered (or cued) to decide to change (e.g., giving up smoking due to coughing or losing weight on a doctor's recommendation).

Mobile technologies have become increasingly prevalent and essential in the delivery of educational content (29). The ubiquity of mobile phones has assisted in overcoming access barriers to adopting and maintaining self-care behaviors (30). Therefore, mobile phones can be effective in educating people about preventing and managing disease and adopting healthy behaviors (29, 31, 32).

Puberty is associated with health risks, and there is a need for effective health education for adolescents that is theoretically informed. Given the fact that mobile technologies are an effective avenue to promote health knowledge and healthy behaviors through health education, it is therefore critically important to evaluate a theory-driven mobile health educational intervention for adolescents based on the HBM. In particular, in Iranian culture, girls are more encouraged to maintain their connection to the family home environment, whereas there is somewhat more tolerance for boys to go unsupervised outside the familial home environment (33). Therefore, this intervention will target the response of boys to the intervention.

## 2 Materials and methods

### 2.1 Study design

The present study was a two-group experimental study (intervention and control groups) that was conducted through WhatsApp and Shad software from 2020 to 2021. The study investigated the efficacy of a mobile-based educational intervention in improving pubertal health behaviors in adolescent boys attending school. The control group received no intervention.

### 2.2 Study setting and sites

Study recruitment sites included schools located in Masjid-i-Sulaiman. Masjid-i-Sulaiman is a city in Khuzestan Province, located in the southwest of Iran.

### 2.3 Participants and sample size

The participants of this study included junior high school boys. To be adequately powered, *N* = 140 students were needed for analyses. After accounting for 10% drop-out, *N* = 156 students (78 interventions and 78 controls) were recruited.

According to geographical divisions, there were four school districts in the city of Masjid-i-Sulaiman. Each district was selected as a cluster, and one school per district (4 schools in total) was randomly selected. The schools were randomized into intervention (*N* = 2) or control (*N* = 2) groups. We randomly divided the schools into intervention and control groups to reduce the possibility of contamination. In the second stage, 39 students were selected by a simple random sampling method from each school. Then, inclusion and exclusion criteria were assessed.

**Figure 1:**
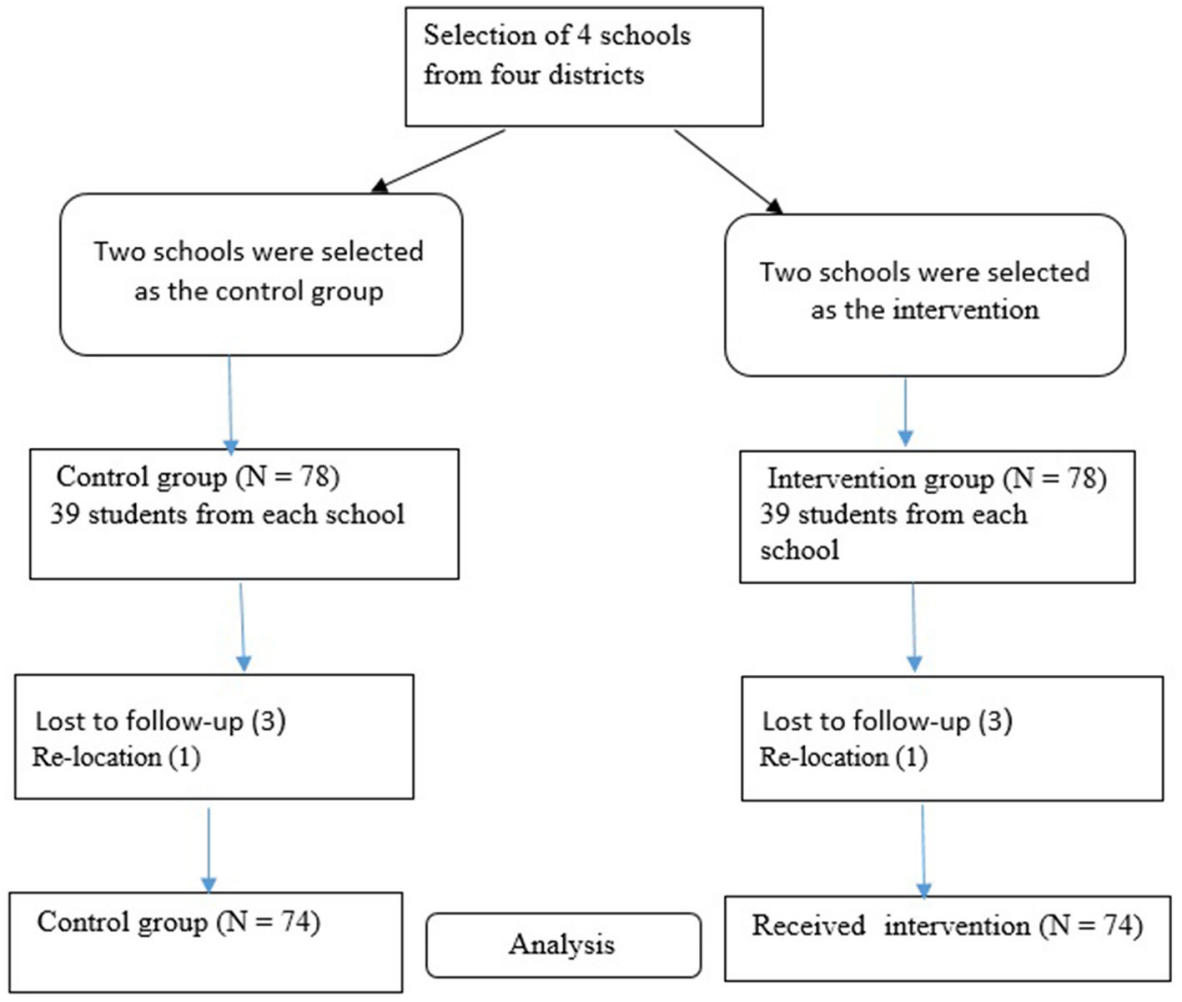
Flow diagram.

Parents provided consent, and the adolescents provided assent. Although *N* = 156 students consented, *N* = 8 boys did not complete the study for various reasons, and a total of *N* = 148 students (*N* = 74 in the intervention group and *N* = 74 in the control group) remained in the study (see Figure 1).

Eighth-grade boys living in Masjid-i-Sulaiman who agreed to the study procedures and had access to WhatsApp and/or Shad social networks were eligible to participate in the study. The **exclusion** criterion of the study was having an underlying disease or disorder that would preclude participation in the study (e.g., active psychosis).

### 2.4 Procedure

The research staff made phone calls to the homes of the potential participants in order to explain the study, and in case they were willing to participate, screening was done and consent was obtained. Baseline questionnaires were completed (via a secure web link) before the intervention started. Boys in the intervention group received an educational program addressing healthy behaviors during puberty based on the HBM (described below) and delivered via WhatsApp or Shad. Shad is a social network and messaging service for Iranian students. Like WhatsApp, Shad is an Internet messenger that provides text and real-time communication (34). The intervention lasted for 4 weeks, and a follow-up assessment was conducted 8 weeks after the intervention was completed. The control group received no intervention, but the questionnaires were yoked with the timing of the intervention group.

### 2.5 Measures

A four-part questionnaire was developed by the research team to assess demographics, health knowledge, HBM constructs, and health behaviors. The initial items of the questionnaire were informed by medical education recommendations according to Iran's Ministry of Health as well as a review of the existing published literature. To enhance the face validity of the tool, seven health promotion, health education, and reproductive health specialists were interviewed in a group to seek their opinions about item content, coverage, clarity, and difficulty. Based on their feedback, items were modified and adjusted.

Next, the content validity ratio (CVR) and content validity index (CVI) were used (35). A panel of eight experts in health education, health promotion, and reproductive health rated the items as follows in terms of relevance: (1) irrelevant, (2) important but not essential, and (3) essential. For each item, CVR was calculated as (n_e_ – N/2)/(N/2), where n_e_ is the number of experts rating the item as essential and N is the number of experts. The overall CVR index of the scale is calculated as the mean of the items' CVR values. To calculate CVI, the experts rated the items on a four-point scale: (1) not relevant, (2) somewhat relevant, (3) quite relevant, and (4) very relevant. CVI is the percentage of experts rating an item as quite relevant or very relevant. The recommended value for CVR is 0.75, and for CVI, the minimum recommended value is 0.79. The final measure was administered to *N* = 20 boys on two occasions, 2 weeks apart, to calculate test-retest reliability. CVR, CVI, and reliability (r) were 0.89, 0.83, and 0.70, respectively. The measure is described below.

Demographic data, including parent education, having a specific illness, etc., were collected (see Table 2). The knowledge section included 11 multiple-choice items (e.g., “Puberty hormones can make a boy's armpit more likely to smell”) with response options of true/false/don't know. “Incorrect” or “I don't know” answers were given 0 points, while correct answers earned 1 point, with the total score ranging between 0 and 11. Higher scores indicate higher levels of knowledge about adolescent health. HBM constructs were also measured. All items were rated on a 5-point Likert scale, from 1 (strongly disagree) to 5 (strongly agree). Perceived susceptibility included five items (e.g., “Probability of academic failure increases if I don't attend to my mental and physical health”), assessing perceived health risks if healthy practices were not maintained during puberty (CVR = 0.87, CVI = 0.86, r = 0.81, total score = 5–25). Perceived severity included six items (e.g., “I may have major health consequences if I don't take care of my hygiene”), assessing the perceived seriousness of poor health should healthy practices not be used during puberty (CVR = 0.83, CVI = 0.91, r = 0.70, total score = 6–30). Perceived benefits included five items (e.g., “By taking care of myself, I can avoid destructive friendships”) assessing perceptions of what might be gained through the use of healthy practices during puberty (CVR = 0.88, CVI = 0.97, r = 0.83, total score = 5–25). Perceived barriers included five items (e.g., “Being shy makes it hard for me to ask questions about puberty”) assessing boys' perceptions of obstacles to performing healthy practices during puberty (CVR = 0.91, CVI = 0.95, r = 0.84, total score = 5–25). Cues to action consisted of seven items (e.g., “Seeing puberty education materials in the mass media makes me want to take action”) assessing stimuli facilitating a decision to employ healthy behaviors during puberty (CVR = 0.92, CVI = 0.97, r = 0.60, total score = 7–35). Finally, self-efficacy consisted of seven items (e.g., “I can follow the principles of personal hygiene”), assessing perceived ability to take action in order to minimize health risks during puberty (CVR = 0.96, CVI = 0.99, r = 0.80, total score = 7–35). Higher scores indicated better status in perceived susceptibility, perceived severity, perceived benefits, self-efficacy, and cues to action, whereas lower scores in perceived barriers indicated a better status.

Health behaviors were assessed based on seven items (e.g., “I regularly eat healthy foods like fruits, and avoid sweets”) rated on a scale from 1 (never) to 5 (always) that reflected healthy behaviors during puberty as endorsed by boys. Higher scores indicate healthier behaviors. CVR = 0.94, CVI = 0.90, r = 0.83, and total score = 7–35 (Supplementary material).

### 2.6 Intervention

Health education, which focused on boys' pubertal development based on HBM, was carried out by a health educator. Five sessions, each lasting 50 min, were presented through WhatsApp and Shad over 4 weeks. As mentioned earlier, Shad is a social network and messaging service for Iranian students, and like WhatsApp, it is an Internet messenger that provides text and real-time communication.

Content included the following topics: exercise, nutrition, sleep, avoiding drugs and alcohol, personal hygiene (bathing, dental care, etc.), sexually transmitted diseases, and information on physiological (e.g., body hair), emotional (e.g., sensation-seeking), social (e.g., peers generally become more important compared to parents), and cognitive (e.g., goal-setting) changes. The sessions were designed to increase puberty knowledge (Session 1) and cover each HBM construct. Session 2 provided information and encouraged exercises to increase perceived susceptibility to risk. Session 3 addressed the serious consequences of not attending to healthy behaviors such as hygiene and exercise (i.e., perceived risk severity), the benefits of healthy behaviors, and perceived barriers to healthy behaviors, along with solutions. Session 4 sought to enhance efficacy in performing health behaviors, and Session 5 determined cues such as reminder texts that encourage healthy behaviors (i.e., cues to action). Methods of delivery included virtual lectures, slides, fact sheets, group discussions, videos, pamphlets, role-plays, and reminder messages. Table 1 provides more detail regarding session number, HBM topic, health content, and method of delivery.

**Table 1:** HBM session content and presentation method.

**Session**	**HBM constructs**	**Topics**	**Method of presentation**
1	Knowledge	The definition of puberty, puberty and associated changes, importance of understanding puberty, healthy behaviors during puberty, time and age of puberty.	Virtual lectures with slides, fact sheet, and group discussion.
2	Perceived susceptibility	Puberty as a time of growth, but also a time of physical, mental health, and social risks (e.g., accidents, sexually transmitted infections, depression, anxiety).	Video presentation, virtual lectures with slides, presentation of statistics, group discussion.
3	Perceived severity	Seriousness of not attending to healthy behaviors (e.g., exercise, nutrition, bathing, choice of friends, etc.) during adolescence. Consequences can be aversive and shorter term (such as embarrassment) or longer term and serious (e.g., life-altering accident).	Audio files, presentation of statistics (e.g., consequences of drug use), group discussion.
3	Perceived benefits	Benefits of performing behaviors to maintain health during puberty (e.g., being strong, saving medical expenses, good mood, positive romantic and peer relations, etc.). Health-behaviors to perform during puberty (e.g., bathing, exercise, nutrition, avoiding sex or protected sex, etc.)	Pamphlets on benefits, group discussion.
3	Perceived barriers	Obstacles to performing pubertal health behaviors (e.g., forgetting to brush teeth). Methods to overcome obstacles.	Group discussion (brainstorming types of barriers), pamphlet on common barriers.
4	Perceived self-efficacy	Teach pubertal health behaviors (e.g., avoid sex, use safer sex, regular bathing, etc.). Understanding setbacks can happen. Stress control methods (e.g., relaxation). Reasons youth should feel confident in their ability to perform healthy behaviors.	Video clips of youth who have successfully performed health behaviors, relaxation training video and practice, role-plays on overcoming barriers, group discussion (brainstorming how to overcome barriers), and testimonials of youth who have overcome barriers.
5	Cues to action	Determine cues that would encourage boys to enact healthy behaviors (e.g., cues in the media, reminder texts), identifying important others who would be proud that boys are taking steps toward responsible adulthood.	Group discussion to identify cues important to each boy (e.g., being a role-model for a younger sibling), visualizing family pride in taking responsibility for health, images depicting boys engaging in healthy behaviors (e.g., avoiding offers for smoking), reminder messages (e.g., stress is normal, but remember, you can use relaxation to help).

Written educational content was divided into several sections, which were provided to students when relevant to facilitate engagement and comprehension. A chat feature was available during sessions for questions and answers. The students were required to activate the audio and video features in their apps. To maintain engagement, the educator sometimes solicits from the participants their thoughts and opinions spontaneously regarding the material presented. Group discussion was encouraged by asking the adolescents to assist their counterparts in problem-solving, brainstorming, etc.

The control group had no intervention, but its questionnaires were also completed online and yoked with the timing of the intervention group. At the conclusion of the study, pamphlets and other educational materials were made available to the control group.

### 2.7 Data analysis

Since the sample sizes in each group (intervention and control) were relatively large, according to the central limit theorem and the law of large numbers, the data were assumed to be normal in each group. The demographic characteristics of the intervention and control groups were summarized using descriptive statistics (e.g., mean, standard deviation, and percentage) and compared using an independent *t*-test and a chi-square test to see if they differed at baseline. As far as health knowledge was concerned, each HBM construct (e.g., self-efficacy) and the health behavior scales were compared at baseline for the intervention and control groups using an independent *t*-test (the same was done in the follow-up). Within the intervention group, each scale at baseline was compared to that at follow-up using paired *t*-tests (the same was done for the control group). Finally, a series of eight ANCOVAs (analysis of covariance) were used to test differences between intervention and control conditions. Dependent variables (DVs) were the knowledge, HBM, and health behavior scales at the 2-month follow-up. Analyses controlled for the baseline score of the DV. Data analyses were performed using SPSS (Statistical Package for Social Sciences; IBM, 2016) version 24 (36), and the significance level was set at 0.05.

### 2.8 Ethics approval

The Ethics Committee of Ahvaz Jundishapur University of Medical Sciences approved the study protocol (IR.AJUMS.REC.1399.635). Participants and their guardians were briefed on the aims and procedures of the study and told that participation was voluntary. The students agreed to participate, and their guardians provided written informed consent for their children to participate.

## 3 Results

The mean age of the participants was 13 years. Table 2 shows the results of the chi-square and *t*-tests, which show no significant difference between the two groups in terms of demographic variables at baseline.

**Table 2:** Description and comparison of demographics by group.

**Demographic**	**Subgroup**	**Control (*N =* 74) N (%) or M (SD)**	**Intervention (*N =* 74) N (%) or M (SD)**	**Total (*N =* 148) N (%) or M (SD)**	**Test statistic (*p*-value)**
Living parents	Both parents	74 (100.0)	72 (97.3)	146 (98.6)	2.03 (0.497)
	Mother	0 (0.0)	2 (2.7)	2 (1.4)	
Living situation	with family	74 (100.0)	73 (98.6)	147 (99.3)	1.01 (1.00)
	in dormitory	0 (0.0)	1 (1.4)	1 (0.7)	
Father's education	Less than a diploma	32 (43.2)	36 (48.6)	68 (45.9)	1.09 (0.578)
	Diploma	37 (50.0)	31 (41.9)	68 (45.9)	
	University	5 (6.8)	7 (9.5)	12 (8.1)	
Mother's education	Less than a diploma	32 (43.2)	38 (51.4)	70 (47.3)	1.06 (0.589)
	Diploma	36 (48.6)	30 (40.5)	66 (44.6)	
	University	6 (8.1)	6 (8.1)	12 (8.1)	
Father's job	Lower class	11 (14.9)	16 (21.6)	27 (18.2)	3.76 (0.288)
	Middle class	16 (21.6)	14 (18.9)	30 (20.3)	
	Upper class	38 (51.4)	29 (39.2)	67 (45.3)	
	No response	9 (12.2)	15 (20.3)	24 (16.2)	
Mother's job	Homemaker	72 (95.9)	69 (93.2)	140 (94.6)	2.31 (0.442)
	Middle class	2 (2.7)	5 (6.8)	7 (4.7)	
	Upper class	1 (1.4)	0 (0.0)	1 (0.7)	
Residence	City	74 (100.0)	74 (100.0)	148 (100.0)	
Financial situation	Difficult	15 (20.3)	18 (24.3)	33 (22.3)	0.38 (0.825)
	Acceptable	42 (56.8)	39 (52.7)	81 (54.7)	
	Good	17 (23.0)	17 (23.0)	29 (19.6)	
	Excellent	0 (0.0)	5 (6.8)	5 (3.4)	
Order of birth in the family	First	39 (52.7)	33 (44.6)	72 (48.6)	5.2 (0.07)
	Second	9 (12.2)	20 (27.0)	29 (19.6)	
	Third and above	26 (35.1)	21 (28.4)	47 (31.8)	
Currently have a physical disease	Yes	1 (1.4)	0 (0.0)	1 (0.7)	1.01 (1)
	No	73(98.6)	74 (100.0)	147 (99.3)	
Number of siblings	Not applicable	4.01 (1.30)	4.20 (1.39)	4.11 (1.34)	0.85 (0.394)

Test statistic is chi-square for categorical and *t-*test for count. M, Mean; SD, Standard Deviation.

Table 3 shows the results of the independent and paired *t*-tests to compare the control and intervention groups in terms of knowledge, health behaviors, and different constructs of the HBM model (i.e., perceived susceptibility, perceived severity, perceived benefits, perceived barriers, self-efficacy, and cue to action). There was no significant difference between the groups at baseline in terms of all constructs except perceived susceptibility (*p*-value = 0.003) and perceived barriers (*p*-value = 0.017). The mean perceived susceptibility and perceived barriers were higher in the control group. At follow-up, all constructs except perceived barriers were significantly higher in the intervention group. Knowledge, perceived susceptibility, perceived severity, perceived benefits, self-efficacy, cue to action, and behavior had an upward trend over the study period in the intervention group, but there was no significant change in the control group. Perceived barriers decreased after the implementation of the educational intervention. All *p*-values were < 0.05.

**Table 3:** Differences between and within groups before and after the health education.

**Health constructs**		**Intervention (*N =* 74) M (SD)**	**Control (*N =* 74) M (SD)**	***T*-test (*p*-value)**
Knowledge	Baseline	5.59 (2.62)	5.91 (2.16)	−0.74 (0.432)
	Follow-up	10.05 (1.25)	6.08 (1.12)	13.86 (< 0.001)^*^
	*T*-test (*p*-value)	−13.38 (< 0.001)	−1.45 (0.150)	
Perceived susceptibility	Baseline	14.24 (2.94)	15.57 (2.31)	−3.04 (0.003)
	Follow-up	19.25 (2.12)	15.53 (2.35)	10.14 (< 0.001)^*^
	*T*-test (*p*-value)	−14.83 (< 0.001)	1.00 (0.321)	
Perceived severity	Baseline	18.35 (2.78)	18.31 (2.66)	0.09 (0.928)
	Follow-up	25.24 (1.823)	18.35 (2.67)	18.34 (< 0.001)^*^
	*T*-test (*p*-value)	−19.02 (< 0.001)	−1.00 (0.321)	
Perceived benefits	Baseline	13.85 (2.27)	13.32 (1.82)	1.56 (0.121)
	Follow-up	18.50 (1.38)	13.37 (1.87)	18.97 (< 0.001)^*^
	*T*-test (*p*-value)	−15.24 (< 0.001)	−1.27 (0.208)	
Perceived barriers	Baseline	14.09 (2.04)	14.91 (2.06)	−2.40 (0.017)
	Follow-up	6.97 (1.66)	14.37 (2.37)	−21.97 (< 0.001)^*^
	*T*-test (*p-*value)	24.95 (< 0.001)	1.89 (0.062)	
Self-efficacy	Baseline	13.59 (1.80)	13.70 (1.79)	−0.37 (0.714)
	Follow-up	18.14 (1.68)	13.80 (1.55)	16.30 (< 0.001)^*^
	*T*-test (*p*-value)	−16.68 (< 0.001)	−1.41 (0.163)	
Cues to action	Baseline	14.34 (2.64)	13.96 (2.79)	0.85 (0.399)
	Follow-up	18.65 (1.49)	14.04 (2.72)	12.76 (< 0.001)^*^
	*T*-test (*p-*value)	−11.92 (< 0.001)	−1.62 (0.109)	
Behavior	Baseline	23.76 (1.60)	21.65 (2.77)	0.85 (0.399)
	Follow-up	29.42 (0.68)	21.65 (2.76)	5.66 (< 0.001)^*^
	*T*-test (*p-*value)	−27.65 (< 0.001)	0.00 (1.000)	

M, Mean; SD, Standard Deviation. ^*^Independent and paired *t*-tests results.

Using ANCOVA, the two groups were compared in terms of DVs, including knowledge, perceived susceptibility, perceived severity, perceived benefits, perceived barriers, self-efficacy, cue to action, and behavior at follow-up, while controlling for the pre-intervention score of the DV. The results in Table 4 show significant differences between the two groups (*p-*values are < 0.05). Compared to the control group, the intervention group experienced a significant increase in knowledge, perceived susceptibility, perceived severity, perceived benefits, self-efficacy, cue to action, and behavior scores and a decrease in perceived barriers. Effect sizes were in the large (and medium) range. The intervention significantly improved the scores on the knowledge, HBM, and health behavior scales at follow-up.

**Table 4:** The effect of the intervention on outcomes controlling for pre-intervention scores.

**HBM constructors**	**SS**	**MS**	**DF**	**F**	***p*-value**	**ES**
Baseline knowledge	108.69	108.69	1	47.100	< 0.001^*^	0.245
Group	614.74	614.74	1	266.394	< 0.001^*^	0.648
Error	334.61	2.31	145			
Baseline perceived susceptibility	312.69	312.69	1	108.50	< 0.001^*^	0.428
Group	692.76	692.76	1	240.38	< 0.001^*^	0.624
Error	417.88	2.88	145			
Baseline perceived severity	293.37	293.37	1	90.68	< 0.001^*^	0.385
Group	1,746.59	1,746.59	1	539.86	< 0.001^*^	0.788
Error	469.11	3.23	145			
Baseline perceived benefits	100.73	100.73	1	49.82	< 0.001^*^	0.256
Group	876.96	876.96	1	433.73	< 0.001^*^	0.749
Error	293.17	2.02	145			
Baseline perceived barriers	470.72	470.72	1	100.54	< 0.001^*^	0.409
Group	781.25	781.25	1	166.87	< 0.001^*^	0.535
Error	678.86	4.68	145			
Baseline self-efficacy	97.22	97.22	1	49.39	< 0.001^*^	0.254
Group	711.45	711.45	1	361.47	< 0.001^*^	0.714
Error	285.39	1.97	145			
Baseline cues to action	262.10	262.10	1	86.05	< 0.001^*^	0.372
Group	719.86	719.86	1	236.34	< 0.001^*^	0.620
Error	441.64	3.05	145			
Baseline behavior	397.42	300.99	1	300.99	< 0.001^*^	0.675
Group	1,178.98	892.92	1	892.92	< 0.001^*^	0.860
Error	191.45	300.99	145			

Effect sizes (ES) in terms of Cohen's f (35): 0.10, 0.25, and 0.40 are small, medium, and large, respectively. DF, Degrees of freedom; MS, Mean sum of square; SS, Sum of squares. ^*^Derived from ANCOVA.

## 4 Discussion

The present study involved an intervention based on the Health Beliefs Model (HBM) and delivered via mobile app in five sessions of 50 min over 4 weeks, which was effective in improving boys' self-reported health behaviors. The results indicate that the intervention improved scale scores over time and improved scores at follow-up compared to the control condition (Table 3). Treatment significantly improved scores of knowledge, HBM scales, and health behavior at follow-up compared to control (Table 4), even after controlling for pre-intervention knowledge, HBM constructs, and behavior. The intervention enhanced the boys' knowledge of how their bodies change during puberty, as well as their perceived susceptibility to health risks and severity, perceived benefits of taking action to reduce risks, self-efficacy to take action effectively, perception of cues that trigger taking action, self-reported health behaviors, and decreased perceived barriers to reduce health risks.

Findings are particularly important since adolescence is a period of significant changes and adolescents are at increased risk for mental health difficulties (2, 4, 6, 7). Forming healthy habits during this period is likely to benefit the individual in the future (2). The intervention is scalable in that it was delivered to 74 students in only five sessions over 4 weeks using mobile technology. It is critical to educate adolescents about the changes associated with puberty to reduce misinformation and health risks that may have long-term consequences (18, 19, 21, 37). The use of the HBM assisted in structuring the intervention by addressing the multiple factors influencing healthy behaviors (26, 27). We note, however, that other models exist to develop health interventions and that they may also be worthy of study to determine their impact on health outcomes.

In the intervention group, the greatest impacts were found in the increased knowledge of changes during puberty and reductions in perceived barriers to enacting healthy behaviors from initial to follow-up assessments. It should be noted that the sample already endorsed substantial engagement in healthy behaviors (a score of approximately 23 out of a possible 35 points). Still, in comparing intervention and control groups, the largest effects were found in the performance of healthy behaviors and perceptions of risk severity, whereas the smallest effects were observed in cues to action and perceived barriers to performing healthy behaviors.

Our results are similar to those of a previous study investigating puberty health education for boys based on HBM, where *N* = 64 boys were randomized to intervention or control (15). Boys in the intervention group experienced an increase in knowledge, perceived susceptibility, perceived severity, perceived benefits, cues to action, and performing health behaviors, while there was no significant improvement in perceived barriers (15, 38). In another study (39), high school boys (*N* = 100) were assigned to the control group or the health education group in order to reduce the risk of the human immunodeficiency virus (HIV) based on HBM. The intervention improved knowledge, perceived susceptibility, perceived severity, perceived benefits, and self-efficacy and reduced perceived barriers. However, cues to action and performance of behavior were not assessed (39). A third pilot study (*N* = 32) on adolescents examined the management of sickle-cell disease based on HBM using a mobile app and measured self-efficacy to manage disease, disease knowledge, and disease-management behaviors (40). From pre- to post-intervention, the participants improved in knowledge and disease-management behaviors. However, there was a variable engagement in the app (40). HBM has wide applicability in its use as a framework to design effective health education programs and has been applied to reducing the risk of skin cancer (41), cancer of the bile duct (42), periodontal disease in pregnant women (43), colorectal cancer (44), adherence to treatment for tuberculosis (45), and controlling hypertension in the older adult (46). Expanding the use of HBM using mobile apps holds promise for improving the health of boys.

Of all the electronic technologies providing health resources, one of the most common services is the provision of health literacy (i.e., knowledge or skill related to health) through mobile phones (47). The results from the current study suggest that adolescent boys improved their pubertal health literacy (i.e., knowledge or skill related to pubertal health) using a common mobile device to receive an intervention based on HBM. The results are consistent with a systematic review (48) showing that e-health literacy (i.e., the ability to find and appraise health information from an electronic source) impacts health-related behaviors. General e-health literacy (i.e., skills needed to use the Internet to understand or apply health information) and HBM constructs (efficacy and perceived benefits) were related to the coronavirus disease-2019 (COVID-19) protective health behaviors, although this study did not examine severity perception or knowledge (49). The results of the current study indicate that HBM constructs (e.g., perceived severity and efficacy) can be directly altered through a carefully designed intervention. A future step may be to determine if HBM constructs mediate the relationship between intervention and puberty health behavior in boys.

### 4.1 Limitations

Future studies should collect a more diverse sample since boys in the current study were generally city-dwelling and healthy, had multiple siblings, and came from two-parent families who were relatively well-educated and financially stable. In addition, with a larger sample size, more schools might be chosen for randomization, allowing more detailed analyses of students within schools, friendship networks, or classrooms. Similarly, a larger sample size might allow for the use of structural equation modeling to examine whether some HBM constructs (e.g., susceptibility or severity) mediate the relationship between cues to action, benefits, self-efficacy, and health behaviors. Although reliance on self-reporting leaves the data vulnerable to inaccuracies, the boys may be in the best position to report on their perceptions. That said, obtaining corroborating reports of boys' health behaviors would improve the methodology in future studies. Another limitation of our study was the lack of standardized instruments in this field. Therefore, we provided valid evidence for the outcome measure used.

Most youths do not receive any intervention related to puberty health at all, so using a no-intervention control has ecological validity. Furthermore, it is reasonable to determine if an intervention has any effect at all—especially against the usual practice (no intervention in this context)—before testing it against a potentially competing intervention. Future studies may seek to test the intervention against another time-matched intervention targeting puberty health.

### 4.2 Strengths, implications, and conclusion

The current study suggests an improvement over earlier works that used smaller sample sizes (e.g., 14 and 39) or that did not measure pivotal constructs including health behavior, cues to action, barriers, or susceptibility (e.g., 47, 55, and 56). In addition, as compared to previous work using mobile app technology (40), the current study had excellent engagement with the apps, perhaps because the participants were given the freedom to choose between two popular apps already in use. This is the first study to examine a scalable mobile phone-based theory-driven health intervention for Iranian boys to improve health behaviors associated with puberty. Given that puberty confers health risks and that there is a need for effective theory-based interventions to address this risk, it is important to deliver this or similar interventions using methods that are readily able to be disseminated. Although this study did not determine if the current program is superior to others in improving boys' health during puberty, it provided evidence indicating that a program based on the Health Beliefs Model (HBM) is relatively efficient and better than no intervention when it comes to improving health behaviors. It is therefore recommended that organizations interested in encouraging healthy behaviors in boys consider the use of such a program since mobile phone education based on the HBM is efficacious in encouraging healthy behavior during puberty in boys.

## Data availability statement

The raw data supporting the conclusions of this article will be made available by the authors, without undue reservation.

## Ethics statement

The studies involving humans were approved by the Ethics Committee of Ahvaz Jundishapur University of Medical Sciences. The studies were conducted in accordance with the local legislation and institutional requirements. The participants provided their written informed consent to participate in this study.

## Author contributions

AS provided the first draft. PR-P conducted the study. MA was the supervisor of the study and designed the study. AS, LS, and SG were the advisors to the study. SG conducted the analyses. All authors helped with the writing and drafting of the manuscript. LS helped with the final editing and proofreading. MA provided the final manuscript. All authors read and approved the final manuscript.
