# Supplementary material for: Using the health beliefs model to implement mobile puberty health education in Iranian adolescent boys: a randomized controlled trial
Source: Front Public Health. 2024 Feb 8;12:1175262. doi: 10.3389/fpubh.2024.1175262 (PMC10882100; doi:10.3389/fpubh.2024.1175262)
Supplement: Supplementary file 1 [file Data_Sheet_1.pdf]

Dear student,
Paying attention to adolescent health is very important since many physical, mental, and social problems as well as unhealthy behaviors are rooted in adolescence. Therefore, the present questionnaire is aimed to determine the factors related to boys' puberty health. Please answer the questions carefully and honestly. Your answers are obviously significant in achieving the desired goals and results of the research. The information obtained will be completely confidential, and the results will be expressed in general terms.
We sincerely thank you for your cooperation.

Part A) Knowledge questionnaire

(Please answer the following questions, and in case you don't know the correct answer, select the "**Don't Know**" option. Please avoid giving random and haphazard answers).

1. What is the first physical change caused by puberty in boys?

1) Enlargement of the testicles

2) Hair growth on the face or armpits

3) Hoarseness of the voice

4) Sudden growth of height and weight

5) I don't know

2. Which of the following factors causes delayed puberty?

1) Excessive obesity or thinness

2) Mental and psychological problems

3) Cold weather

4) All of above

5) I don't know

3. When does a boy have late puberty?

1) Physical and sexual signs of puberty do not appear until the age of 14.

2) The physical and sexual signs of puberty do not appear until the age of 16.

3) Physical and sexual signs of puberty do not appear until the age of 18.

4) Physical symptoms appear until the age of 14, but sexual symptoms do not appear until 2 years later

5) I don't know

4. When is a boy matured?

1) With intellectual and social development

2) With physical and physical development

3) Ejaculation during sleep

4) All of above

5) I don't know

5. At what time are mood swings, anger, and aggression more evident?

1) Pre-puberty period

2) Puberty period

3) Post-puberty period

4) There is no specific time.

5) I don't know

6. Which of the following is a symptom of depression in teenagers?

1) Indifference towards everything and anyone

2) They do not enjoy anything and sometimes cry

3) They don't have the relationships with their friends as they used to

4) All of above

5) I don't know

7. What are the main food groups that should be included in the adolescent diet?

1) Meat, beans and nuts

2) Milk, yogurt, and dairy products

3) Fruits and vegetables

4) All of above

5- I don't know

8. What effect can regular sports activities have on a person's physical and mental health during puberty?

1) Satisfaction with physical appearance and increasing self-confidence

2) Reducing depression, anxiety, tension, and worry

3) Boosting high spirits and expending energy

4) All of above

5) I don't know

9. What recreations during puberty and adolescence can be unhealthy and risky for the individual?

1) Participating in unhealthy friend activities

2) Being too busy with the Internet and unauthorized social networks

3) Arbitrary use of pills and medicine advised by friends

4) All of above

5) I don't know

10. Which of the following personal hygiene points is important to observe during puberty?

1) Using personal hygiene items (scissors, razors, etc.) to remove unwanted hair.

2) Proper sleep and rest during the day and night

3) Regular and correct brushing of teeth

4) All of above

5) I don't know

11. How should face pimples be treated properly?

1) Washing the pimples every day with soap and water

2) Using medical alcohol to disinfect the site of pimples

3) Avoiding manipulating and pressing the pimples

4) All of above

5) I don't know

Part B) Questionnaire of the constructs of the health belief model

Please indicate your opinion on each of the following questions by marking ✓ or **x** from completely agree to completely disagree in the corresponding column.

All items are rated on a 5-point Likert scale (strongly disagree (1), disagree (2), have no opinion (3), agree (4) a completely agree (5)).

Items	**Perceived susceptibility**
1	Sudden physical and mental fluctuations and changes during puberty affect my health.
2	In my opinion, associating with unhealthy friends and participating in their activities affects my health.
3	Like my friends, I may experience academic failure during puberty.
4	I think that I will have problems with my mood when puberty begins.
5	In my opinion, lack of self-control during adolescence causes sexually transmitted diseases such as AIDS and hepatitis.
	**Perceived severity**
1	Failure to observe personal hygiene during puberty may have dangerous consequences for me.
2	Participating in activities of unhealthy friends is considered a serious risk for me.
3	Not exercising during puberty will be problematic for me.
4	The rapid changes of puberty cause anxiety and stress in me.
5	Smoking cigarettes and hookah and taking illegal drugs and pills can cause dangerous side effects and even death for me in the future.
6	Taking illegal pills and powders to make my body muscular is a serious risk for me.
	**Perceived benefits**
1	By taking care of myself, I can avoid destructive friendships.
2	It will be useful for me not to associate with unhealthy friends during puberty.
3	Adherence to proper nutrition during puberty will cause proper physical growth.
4	Exercising during puberty prevents physical and mental problems.
5	Increasing awareness of puberty issues by the family will help me get through puberty issues easily.
	**Perceived barriers**
1	I am embarrassed to ask my family members questions about puberty issues.
2	I am embarrassed to ask my questions about maturity issues from school teachers.
3	If I don't join my friends in unhealthy entertainment activities, they will make fun of me and leave me alone.
4	Because I do not have information about puberty issues, I cannot make a correct decision about the problems that arise in this regard.
5	Due to economic problems in the family, I do not have proper nutrition.
	**Self-efficacy**
1	I can observe the principles of personal hygiene during puberty.
2	I am sure that I can get correct information about puberty from reliable sources.
3	I am sure that I can consult with a doctor or a counselor in case of problems related to puberty.

4	I am confident that I can help to improve physical and mental maturity with appropriate sports activities.
5	I am confident that I can have a suitable diet plan to improve puberty function.
6	I am sure that I can avoid smoking.
7	I am sure that I can observe my sexual hygiene during puberty.
**Cues to action**	
1	I learned about the characteristics of this period with the information I received from my friends at school about the changes of puberty.
2	I receive the necessary information and cues regarding puberty from my family.
3	I receive the necessary information and cues regarding puberty and its changes from health centers.
4	I receive puberty education from mass media (radio, television, etc.).
5	Through reading books and magazines, I get to know the changes of puberty.
6	Seeing educational materials related to puberty on the Internet and mass media makes me want to take action.
7	7I receive the necessary information about puberty care from the school health care provider.

Part C) Questionnaire of the Health behaviors

All items are rated on a 5-point Likert scale (always (5), often (4), sometimes (3), rarely (2), never (1)).

Items	**Health behaviors**
1	I refrain from smoking cigarettes and hookah.
2	I regularly eat healthy foods such as fruits and avoid sweets.
3	I have at least half an hour of regular exercise every day.
4	I avoid manipulating my pimples and wash my face with warm water and soap every day.
5	I bathe regularly.
6	I use personal hygiene items such as razors for hair removal and personal hygiene.
7	I refuse to take illegal pills and powders advised by friends to make my body muscular.
